# Programmed Cell Death-Dependent Host Defense in Ocular Herpes Simplex Virus Infection

**DOI:** 10.3389/fmicb.2022.869064

**Published:** 2022-04-08

**Authors:** Hongyan Guo, Heather S. Koehler, Richard D. Dix, Edward S. Mocarski

**Affiliations:** ^1^Department of Microbiology and Immunology, Louisiana State University Health Sciences Center Shreveport, Shreveport, LA, United States; ^2^Department of Microbiology and Immunology, Emory University School of Medicine, Atlanta, GA, United States; ^3^School of Molecular Biosciences, College of Veterinary Medicine, Biotechnology Life Sciences, Pullman, WA, United States; ^4^Viral Immunology Center, Department of Biology, Georgia State University, Atlanta, GA, United States; ^5^Department of Ophthalmology, Emory University School of Medicine, Atlanta, GA, United States

**Keywords:** apoptosis, necroptosis, pyroptosis, herpes simplex virus, ocular infection

## Abstract

Herpes simplex virus type 1 (HSV1) remains one of the most ubiquitous human pathogens on earth. The classical presentation of HSV1 infection occurs as a recurrent lesions of the oral mucosa commonly refer to as the common cold sore. However, HSV1 also is responsible for a range of ocular diseases in immunocompetent persons that are of medical importance, causing vision loss that may result in blindness. These include a recurrent corneal disease, herpes stromal keratitis, and a retinal disease, acute retinal necrosis, for which clinically relevant animal models exist. Diverse host immune mechanisms mediate control over herpesviruses, sustaining lifelong latency in neurons. Programmed cell death (PCD) pathways including apoptosis, necroptosis, and pyroptosis serve as an innate immune mechanism that eliminates virus-infected cells and regulates infection-associated inflammation during virus invasion. These different types of cell death operate under distinct regulatory mechanisms but all server to curtail virus infection. Herpesviruses, including HSV1, have evolved numerous cell death evasion strategies that restrict the hosts ability to control PCD to subvert clearance of infection and modulate inflammation. In this review, we discuss the key studies that have contributed to our current knowledge of cell death pathways manipulated by HSV1 and relate the contributions of cell death to infection and potential ocular disease outcomes.

## Introduction

Herpes simplex virus 1 (HSV1) is a neurotropic double-stranded DNA virus belonging to the alpha subfamily of the *Herpesviridae* family (Fields et al., 2013). The virus has long been recognized as one of the most well-adapted human pathogens, infecting an overwhelming majority of the world’s population (Smith and Robinson, 2002; Whitley et al., 2007; Koujah et al., 2019). Following primary infection at peripheral sites, the virus travels neural circuitry of the peripheral nervous system to gain access to neurons of sensory ganglia where the virus establishes a life-long latent infection (Sodroski, 2021). On occasion, the virus will invade the central nervous system, causing a life-threatening encephalitis (Bradshaw and Venkatesan, 2016). This pattern of pathogenesis has raised the controversial possibility that HSV1 triggers of chronic neurological disorders such as Alzheimer’s disease (Marcocci et al., 2020).

The most common clinical manifestation associated with HSV1 infection in immunologically normal persons is oral disease that typically presents as recurrent bouts of cold sores that originate from virus that reactivates from latency in trigeminal ganglia (Whitley and Roizman, 2001). Less frequently, HSV1 causes ocular diseases associated with significant vision loss and blindness worldwide (Liesegang, 2001). These range from recurrent corneal disease called herpes stromal keratitis (HSK) to acute retinal necrosis (ARN), a rare sight-threatening disease that destroys the retina (Kanukollu and Patel, 2021). While the clinical features of HSV1-associated ocular diseases have been well characterized, the events that contribute to the onset and development of these diseases are just being characterized. In this review, we explore the possibility that programmed cell death pathway-dependent host defenses play a heretofore unrecognized yet significant role either individually or collectively during the pathogenesis of HSV1-mediated corneal and retinal diseases.

## HSV1 Replication Cycle

The mature HSV1 virion carries a large (>150 kilobase pair) double-stranded DNA genome encased in an icosadeltahedral protein capsid surrounded by a tegument layer consisting primarily of viral proteins and encased in a lipid bilayer envelope that incorporates virus-encoded glycoproteins, including gB, gC, gD, gH, and gL, that orchestrate viral entry into cells (Farooq et al., 2010). HSV1 envelope glycoproteins interact with cell surface receptors and drive membrane fusion that deposits the nucleocapsid in the cytoplasm. HSV1 entry may occur directly at the cell surface *via* a membrane fusion pore or from an intracellular vesicle following endocytic or phagocytic uptake (Clement et al., 2006). Initial contact of viral glycoproteins gB and/or gC with heparan sulfate proteoglycan on the cell surfaces (Shukla and Spear, 2001) leads to binding of gD to a host receptor, such as nectin-1 or herpes virus entry mediator (HVEM) and facilitated by 3-O-sulfated heparan sulfate (3-OS-HS), that eventually triggers a conformational change in gB, in concert with a gH/gL complex to mediate membrane fusion (Carfi et al., 2001; Shukla and Spear, 2001). In the process, gB undergoes a conformational change from pre-fusion to post-fusion after binding to a receptor such as immunoglobulin-like 2 (PILRα), n-muscle myosin heavy chain IIA (NMHC-IIA) or myelin-associated glycoprotein (MAG). Once inside the cytoplasm, the viral nucleocapsid translocates using cellular microtubule-associated movement to a nuclear pore where uncoating and release of HSV1 DNA into the nucleus becomes complete and replication ensues (Sodeik et al., 1997; Dohner et al., 2002).

Once viral DNA has been delivered to the nucleus, viral gene expression commences in a highly regulated cascade. Viral genes are grouped into at least three categories (immediate early, delayed early, and late, also termed by the Greek symbols a, b, and g, respectively) based on long-established temporal expression patterns (Whitley and Roizman, 2001). In the first stage of viral gene expression, a multiprotein complex orchestrated by a viral tegument protein, VP16, together with cellular proteins, such as Oct-1 and HCF-1, recruit RNA polymerase II specifically to the immediate early (IE or a) gene promoters (Campbell et al., 1984; Gerster and Roeder, 1988; Kristie and Sharp, 1993) controlling expression of infected cell protein (ICP) 0, 4, 22, 27, and 47 (Honess and Roizman, 1974). The IE genes in turn promote the expression of additional, early (b) and late (g) viral genes (Roizman and Zhou, 2015). IE genes expression peaks between 2 and 4 h after infection; early genes are transcribed independent of viral DNA replication, peaking between 6 and 12 h post-infection; and late gene expression peaks between 10 and 16 h post-infection (Ibanez et al., 2018). Curiously, in all herpesviruses, including HSV1, there are two categories of late gene expression, one proceeding independent of viral DNA synthesis (so called leaky late or g1 genes) and the other only detected once viral DNA synthesis starts (so called true late or g2 genes).

## Overview of Apoptotic Cell Death Signaling

Apoptosis is a programmed cell death pathway employed by all multicellular organisms to eliminate excess, aging or stressed cells during development and homeostasis (Cookson and Brennan, 2001; Sakamaki et al., 2002). Apoptosis contributes to host defense by eliminating infected cells and preventing spread of intracellular pathogens within the host animal (Feng et al., 2019). Apoptosis depends on a caspase-dependent proteolytic cascade that coordinates characteristic cell-membrane blebbing, nuclear condensation and DNA fragmentation, while maintaining membrane integrity (Hengartner, 2000; Strasser et al., 2000). Depending on the nature of stress or death trigger and on the initiator caspase that becomes activated, apoptotic death proceeds *via* either extrinsic or intrinsic pathways (Nagata, 1997; Green, 1998; Sun et al., 1999). The intrinsic apoptosis is triggered by signals originating within the cell that arise from viral infection such as DNA damage, endoplasmic reticulum stress, cell cycle dysregulation or oxidative stress (Green and Evan, 2002). Intrinsic apoptosis depends on signaling events transmitted *via* mitochondria that result in mitochondrial outer membrane permeabilization as a key step that mediates the execution of cells. The balance of pro-apoptotic B cell lymphoma 2 (BCL2) family members BAX and BAK working in concert with additional pro- as well as anti-apoptotic BCL2 family proteins dictate cell fate. Following release from mitochondria, mediators such as cytochrome *c* (Danial and Korsmeyer, 2004), which forms a complex with Apaf1, activate caspase-9 and downstream effector caspases such as caspase-3 and caspase-7 that results in cell death (Green, 1998; Sun et al., 1999).

Extrinsic apoptosis depends upon specific self-activating caspases, caspase-8 (conserved in mice and humans) and caspase-10 (in humans). Best studied downstream of death receptor (TNFR1, FAS or TRAIL) signaling (Ashkenazi and Dixit, 1998) where a receptor death domain (DD) recruits downstream signaling adaptors to drive formation of a multi-component ripoptosome (Mocarski et al., 2011). Autocleavage activation of procaspase-8 leads to activation of effector caspases (caspase-3 or -7) either directly or with the cooperation of BCL2 family proteins that amplify signals *via* mitochondrial steps similar to intrinsic apoptosis. Thus, diverse cell-intrinsic and cell-extrinsic initiators converge on the activation of executioner caspases, which cleave cellular proteins important for cell maintenance such as the DNA repair enzyme, poly(ADP-ribose) polymerase (PARP) (Kaufmann et al., 1993; Lazebnik et al., 1994), structural elements, lamin B and actin (Lazebnik et al., 1995; Mashima et al., 1995), and the DNase inhibitor DNA fragmentation factor 45, DFF-45 (Liu et al., 1997). The cleavage of these cellular protein targets by the executioner caspases drives the cellular death and is responsible for the hallmark morphological and biochemical changes characteristic of apoptosis.

## HSV1 Manipulation of Apoptosis Pathways

Cells infected with HSV1 do not show typical features of apoptosis, such as fragmentation of chromosomal DNA into nucleosome oligomers or characteristic morphological changes (Koyama and Miwa, 1997). However, HSV1 infection of human Hep2 cells in the presence of a protein synthesis inhibitor, cycloheximide (CHX), results in apoptosis (Koyama and Adachi, 1997), suggesting that newly synthesized viral proteins actively prevent this pathway during productive infection of susceptible cells. Initial identification of HSV1-encoded cell death suppressors was based on the induction of cell death during infection with mutant strains of virus. The HSV1 ICP4-deficient mutant (d120), which lacks both copies of this IE gene, seemed to induce apoptosis (Leopardi and Roizman, 1996); however, a follow-up study failed to show ICP4 alone was sufficient to block apoptosis (Leopardi et al., 1997). Instead, this line of investigation unveiled US3, a late gene encoding a serine/threonine kinase as a cell death suppressor somehow working together with UL13 (Eaton et al., 2014), another gene encoding a protein kinase. Low levels of US3 expression were responsible for the susceptibility of d120 mutant virus to apoptosis (Leopardi et al., 1997). One possibility could be that US3 is acting as a viral kinase to mimic the host AKT1 and restrict apoptotic signaling thereby enhancing viral translation and replication (Chuluunbaatar et al., 2010). Although protein phosphorylation influences apoptotic signaling and both US3 and UL13 have a range of purported activities, the precise mechanisms through which virus-encoded protein kinases prevent apoptosis still needs more exploration.

Additional studies implicated another IE gene product, ICP27, a critical viral function supporting replication, in suppressing apoptosis. ICP27-deficient virus (vBSΔ27) induced death in three different human cell lines (Hep2, HeLa, and 143TK^–^) (Aubert and Blaho, 1999). Infections with this mutant virus in Vero cells that are known to be deficient in interferon signaling failed to drive apoptosis (Aubert and Blaho, 1999), suggesting that ICP27 may restrict apoptosis by interfering with interferon pathways.

This anti-apoptosis behavior was further evaluated by comparing virus-induced apoptosis to other inducers, such as tumor necrosis factor (TNF), antibody to Fas, C2-ceramide, osmotic shock (sorbitol), and thermal shock (Galvan and Roizman, 1998). In addition to protecting viral infection-induced apoptosis, wildtype HSV1 infection protected human neuronal SK-N-SH cells from death induction by pro-apoptotic agents.

ICP22, another IE protein, has also been implicated in preventing apoptosis of HSV1-infected human Hep2 cells (Aubert et al., 1999). Moreover, Late genes, gD and gJ (encoded by US6 and US5 genes, respectively), independently contribute to restricting virus-induced apoptosis in human T and neuroblastoma cells (Jerome et al., 1999; Zhou et al., 2000).

**FIGURE 1:**
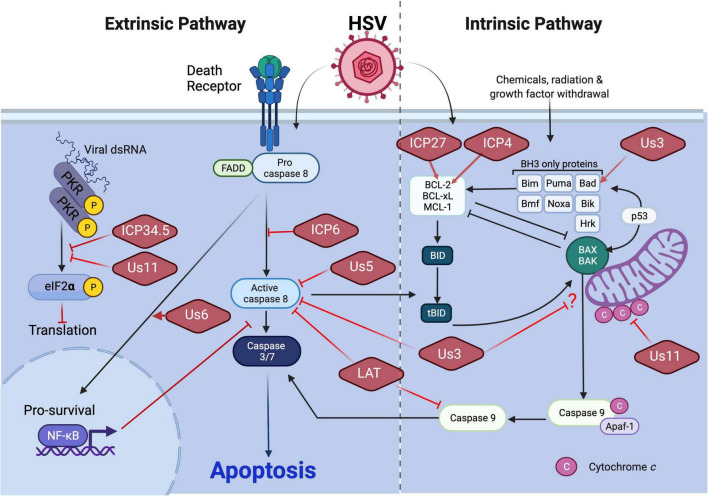
HSV1 manipulation of apoptosis. Execution of apoptosis restricts HSV1 replication and dissemination. The extrinsic and intrinsic apoptosis pathways differ with respect to the nature of the death signal as well as initiator caspase (see text). HSV1 encodes an array of anti-apoptotic viral proteins to counteract apoptotic pathways. Virus-encoded ICP34.5 or Us11 limits the extrinsic apoptosis pathway by restricting dsRNA sensing pathways that would lead to activation of apoptosis through inhibition of protein translation. Virus-encoded ICP6 or Us5 block extrinsic apoptosis by preventing caspase 8 activation. Virus-encoded Us6 promotes NF-kB-dependent pro-survival signaling. Virus-encoded Us3 blocks both extrinsic and intrinsic apoptosis through interference with caspase 8 activity and an unknown step in mitochondrial death signaling, respectively. Us3 has also been shown to promote the expression of Bad to limit intrinsic apoptosis as does Us11 through restriction of cytochrome *c* release from mitochondria. Expression of the pro-survival BCL-2 gene of the intrinsic pathway also has been shown to be stimulated by virus-encoded ICP27 or ICP4. Finally, LAT manipulates the activation of both the extrinsic and intrinsic apoptosis pathways specifically within the central nervous system by blocking inducer caspases 8 and caspase 9, respectively.

These studies revealed the capacity of HSV to prevent apoptosis most likely at different stages in the replication cycle (Figure 1), although most of the studies relied on observations with various HSV1 mutants in susceptible human cell types, some of which are unlikely to be involved in natural settings. Transfected gJ partially protected T cells from Fas or granzyme B-mediated apoptosis (Jerome et al., 2001). Anyway, whether these reported antiapoptotic viral genes alone are sufficient to protect cells from other stimuli-induced apoptosis in non-species manner remains to be fully elucidated.

In addition to antiapoptotic genes described above, the latency associated transcript (LAT) suppresses cell death and contributes to efficient reactivation (Perng et al., 2000). The antiapoptotic function of LAT was first recognized using LAT-null mutant derived from the McKrae strain of HSV1 (dLAT2903). The TG of rabbits infected with dLAT2903 showed higher levels of apoptotic cells compared to wild-type parent virus (Perng et al., 2000). LAT is notable for its ability to block exogenously induced intrinsic apoptosis in both human and mouse neuronal cells (Perng et al., 2000; Inman et al., 2001; Ahmed et al., 2002). LAT control of apoptosis has been confirmed additional strains of virus as well as various animal models (Branco and Fraser, 2005). Expressed LAT in murine neuronal cells in the absence of other viral genes is sufficient to inhibit both the caspase-8 and caspase-9 activation, indicating LAT acts upstream of these proteases (Figure 1; Henderson et al., 2002). Although the mechanism of apoptosis interruption is still unclear, evidence suggests that LAT may indirectly affect apoptosis *via* silencing innate immune signaling pathways (Li et al., 2010; Carpenter et al., 2015; Tormanen et al., 2019).

ICP6 is the best understood cell death suppressor encoded by HSV1 or HSV2. As an early protein, ICP6 functions as a component of the large subunit of ribonucleotide reductase (denoted RR1) responsible with RR2 for conversion of ribonucleoside diphosphates to the corresponding deoxyribonucleotides to ensure adequate precursor pools for synthesis of viral DNA. Studies with ICP6-deficient HSV1 affirmed that this enzyme supports efficient replication in non-dividing cells where deoxyribonucleotide pools are limiting (Goldstein and Weller, 1988a,b). In addition to its role in nucleotide metabolism, ICP6 from either HSV1 or HSV2 is sufficient to inhibit activation of caspase-8 induced by TNF or Fas ligand in human cells (Figure 1). Importantly, this viral protein acts specifically on extrinsic apoptosis and does not block cell death mediated by the mitochondrial pathway induced by Bax overexpression, etoposide, staurosporine or menadione (Langelier et al., 2002). The mechanism of extrinsic apoptosis suppression mediated by ICP6 has become the best understood for this type of virus.

The large C-terminal enzymatic domain of ICP6 exhibits direct interaction with caspase-8 death effector domain (DED) along with the ability to prevent FADD recruitment of caspase-8, which is necessary for extrinsic apoptosis to proceed (Dufour et al., 2011b). ICP6 also protects cells from TLR3-mediated apoptosis triggered by double-stranded RNA (dsRNA) *via* directly interacting caspase-8 and receptor-interacting protein kinase (RIPK) 1, indicating an ability of this viral protein to target signaling components in addition to caspase-8 (Dufour et al., 2011a). The interaction between ICP6 and RIPK1 contributes to suppression of an alternative cell death pathway, necroptosis, as elaborated below.

## Overview of Necrotic Cell Death Signaling

In the course of the past 20 years, necrosis has progressed from being considered a dysregulated or incidental (passive) route to tissue damage with the characterization of a programmed necrotic death pathway called necroptosis (Mocarski et al., 2011). Much of the delay in recognizing necroptosis as a bonified PCD pathway can be attributed to the preponderance of isolated cells utilized in culture that repress necroptosis signaling through epigenetic regulation of RIPK3 (Koo et al., 2015). With the identification of cells that retrain the expression of the essential necroptosis machinery, it is now recognized this PCD pathway is a highly regulated cell-autonomous pathway that occurs when caspase-8 function is compromised or absent (Mocarski et al., 2011). Necroptosis uniformly relies on RIP homotypic interaction motif (RHIM)-dependent recruitment of RIPK3, a protein kinase whose activity is essential for the pathway to play out. Necroptosis may be initiated by RIPK1 (Cho et al., 2009; He et al., 2009; Zhang et al., 2009) downstream of death receptor signaling, TIR-domain-containing adapter-inducing interferon-β (TRIF) downstream of Toll-like receptor-3 or -4 (He et al., 2011) or Z-nucleic acid (Z-NA) binding protein 1 (ZBP1, also called DAI) a pathogen sensor of Z-NA (Upton et al., 2012; Thapa et al., 2016; Koehler et al., 2017; Sridharan et al., 2017; Guo et al., 2018; Zhang et al., 2020). Once triggered by interaction with RIPK1, TRIF, or ZBP1, RIPK3 acts to phosphorylate itself and downstream executioner mixed lineage kinase domain-like (MLKL) to execute cell death (Sun et al., 2012). In contrast to apoptosis, necroptosis is caspase-independent and involves cell rounding and cytoplasmic swelling, terminating with the loss of membrane integrity and cell leakage (Yuan and Kroemer, 2010).

The potency and delicate balance of apoptosis and necroptosis pathways was first brought to light in studies that revealed a RIPK1–RIPK3-dependent process dictating midgestational embryonic lethality of caspase-8-deficient mice (Kaiser et al., 2011; Oberst et al., 2011) or FADD-deficient mice (Zhang et al., 2011). This was further reinforced when RIPK1 deficiency was shown to unleash a combination of caspase-8 and RIPK3-mediated pathways that are normally held in check *via* RIPK1 RHIM signaling through the final stages of gestation and during parturition, a time during development when tonic levels of TNF, interferons, and nucleic acids combine to regulate necroptosis signaling through the ripoptosome (Dillon et al., 2014; Kaiser et al., 2014; Rickard et al., 2014).

A ripoptosome requires collaboration of FADD, caspase-8, cFLIP_*L*_, RIPK1, or RIPK3 and forms through DED-dependent, DD-dependent, and RHIM-dependent interactions (Feoktistova and Leverkus, 2015). The propensity for a ripoptosome to dictate apoptotic or necrotic outcomes emerged from studies of RIPK3 mutant-bearing cells, mice and the behavior of RIPK3 kinase inhibitors, where viability of cells and mice was undermined by RHIM dependent signal transduction associated with robust ripoptosome formation (Mandal et al., 2014; Newton et al., 2014).

## HSV1 Manipulation of Necroptosis Pathways

**FIGURE 2:**
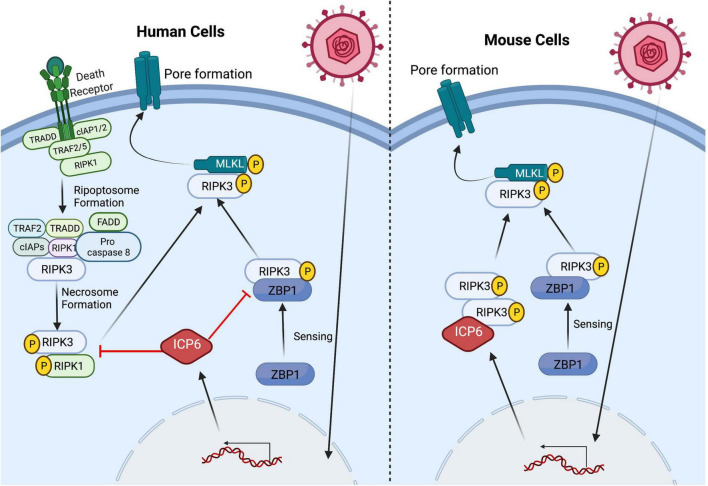
HSV1 manipulates the necroptosis pathway in a species-specific manner. Triggering of necroptosis *via* several upstream signaling pathways is dependent on the activation of the RHIM-containing protein RIPK3 which, in turn, mediates MLKL oligomerization and translocation to the plasma membrane resulting pore formation and ultimately cell death (see text). HSV1 manipulates the necroptosis pathway through virus-encoded ICP6 RHIM-mediated interaction with RHIM-containing host proteins, but the outcome of this interaction results in species-specific outcomes. In HSV1-infected human cells, ICP6 acts as a RHIM competitive inhibitor and restricts necroptosis by sequestering RIPK3 from its cellular partners ZBP1 and RIPK1. In contrast, ICP6 expression in HSV1-infected mouse cells results in ICP6 RHIM dependent recruitment of RIPK3 that drives necroptosis. HSV1 infection triggers RIPK3-mediated necroptosis *via* ZBP1 sensing z-form RNA in both human and mouse cells.

Necroptosis emerged as antiviral host defense mechanism from studies on murine cytomegalovirus (MCMV) infection of its natural mouse host. When the RHIM domain of M45 (a homolog of HSV1 ICP6) was mutated, M45 failed to block RHIM-dependent signaling (Upton et al., 2010, 2012). During infection of susceptible cells or mice, M45mutRHIM MCMV triggers ZBP1-mediated necroptosis, cutting short replication and restricting dissemination (Upton et al., 2010). HSV UL39-encoded ICP6, like the MCMV M45-encoded RR1 homolog, binds RIPK1, potentially *via* its RHIM as well as through another interaction region (Dufour et al., 2011a). As predicted (Lembo and Brune, 2009), HSV ICP6 carry a similar N-terminal RHIM that mediates binding to RIPK1 and RIPK3 (Wang et al., 2014; Guo et al., 2015; Huang et al., 2015). In human cells, this interaction prevents RIPK3 activation and formation of a necrosome downstream of TNFR1- and Fas-dependent necroptosis (Guo et al., 2015). In contrast to human cells, ICP6 induces necroptosis in mouse cells. This species-specific function of ICP6 appears to be independent of death receptor, ZBP1 or TRIF signaling (Wang et al., 2014; Huang et al., 2015; Figure 2). Similar interactions of ICP6 appear to block necroptosis in human cells yet trigger necroptosis in mouse cells dependent on the impact of this particular RHIM because substitution of MCMV M45 RHIM converted HSV1 ICP6 into an anti-necroptotic function in mouse cells (Huang et al., 2015).

The C-terminal domain of ICP6 known to control apoptosis is also necessary for suppression of necroptosis in human cells. Neither M45 nor human CMV UL45 bind to caspase-8 (Guo et al., 2015). Both betaherpesviruses encode the separate caspase-8-binding protein, viral inhibitor of caspase-8-induced apoptosis (vICA). Thus, in addition to blocking caspase-8-dependent apoptosis, the RNR domain of HSV1 ICP6 simultaneously opens the pro-necrotic trapdoor in human cells by blocking caspase-8 activation while also blocking this alternate outcome *via* RHIM-dependent disruption of RIPK1–RIPK3 interaction.

ZBP1 was implicated as a pathogen sensor capable of recognizing HSV1 DNA in mouse cells, along with cyclic GMP–AMP synthase (cGAS) and stimulator of interferon genes (STING) (Takaoka et al., 2007; Sun et al., 2013). ZBP1 functions as crucial adaptor for RHIM-dependent activation of RIPK3-dependent necroptosis during MCMV (Upton et al., 2012) infection and has more recently been implicated in the induction of necroptosis by influenza (Thapa et al., 2016) as well as vaccinia virus (VACV) (Koehler et al., 2017). Most of the accumulated evidence indicates that ZBP1 senses the accumulation of RNA rather than DNA to initiate RHIM exposure and recruitment/activation of the RIPK3. This sensing occurs during herpesvirus, poxvirus and orthomyxovirus infections (Sridharan et al., 2017; Zhang et al., 2020; Koehler et al., 2021). Further studies revealed a species specificity in the function of HSV1 ICP6 which blocks necroptosis in human cells but directly activates RIPK3 *via* RHIM interactions in mouse cells (Guo et al., 2018). HSV1 ICP6 RHIM mutant virus triggers ZBP1/RIPK3/MLKL-dependent necroptosis in both mouse and human cells, thereby establishing the role of ZBP1 as an evolutionarily conserved sensor of HSV1 in both species (Guo et al., 2018; Figure 2). Importantly, HSV1 transcription is required for initiation of necroptosis independent of input viral DNA or replication dependent amplification of viral DNA (Guo et al., 2018). These observations expand the number of examples where necroptosis is induced *via* this Z-NA sensor. Furthermore, the increased susceptibility of ZBP1-deficient mice to ICP6 mutant virus clearly demonstrates an antiviral role for ZBP1 independent of other, species-specific characteristics of this virus (Guo et al., 2018).

## Overveiw of Pyroptosis

Like necroptosis, pyroptosis is a lytic, inflammatory type of programmed cell death. This necrotic type of cell death was first described in 1992 (Zychlinsky et al., 1992), but the term was coined in 2001 following the observation that bacteria-infected macrophages underwent a rapid necrotic cell death dependent on caspase-1 activity (Cookson and Brennan, 2001). Although traditionally defined as caspase-1-mediated cell death, studies have revealed other caspases, caspase-11 in mice and orthologs caspase-4 and -5 in humans (Kayagaki et al., 2011; Shi et al., 2014), and more recently the apoptotic effector caspase, caspase-3, as being capable of triggering pyroptosis (Rogers et al., 2017; Wang et al., 2017). The execution of pyroptotic cell death *via* these caspases is a result of their ability to cleave and activate specific members of the pore-forming gasdermin gene family (Shi et al., 2015; Ding et al., 2016). Gasdermin N- and C terminal linker domain cleavage releases an activated N terminal region from an inhibitory C-terminal fragment (Shi et al., 2015; Ding et al., 2016). The gasdermin-N domain binds to acidic phospholipids, such as phosphoinositides found on the inner leaflet of the mammalian plasma membrane, to form oligomeric death-inducing pores (Aglietti et al., 2016; Ding et al., 2016; Liu et al., 2016; Sborgi et al., 2016). In the canonical model of caspase 1-mediated pyroptosis, recognition of inflammatory ligands leads to activation of intracellular multiprotein signaling complexes known as the inflammasomes. Among the best studied inflammasome sensors are absent in melanoma 2 (AIM2), Pyrin, and the NOD-like receptor (NLR) family members (NLRP1, NLRP3, and NLRC4) (Frank and Vince, 2019). Upon activation, caspase 1 is recruited to the inflammasome sensor proteins *via* the CARD-domain containing adaptor protein (ASC) which triggers auto-processing of the inactive form of caspase-1 to its catalytically active species, p46 and p33/p10 subunits (Boucher et al., 2018). Activated caspase-1 cleaves and activates GSDMD, as well as the inflammatory cytokines, IL-1β and IL-18, which are critical mediators in host innate immune responses against various pathogens. In contrast to caspase-1, non-canonical inflammasomes are defined by their requirement for caspase-4/5/11, which have been reported to directly bind cytosolic LPS, resulting in their targeting and activation of GSDMD (Shi et al., 2014). Although caspase-4/5/11 do not process IL-1β and IL-18 directly, their activity does cause GSDMD-mediated potassium efflux, which suffices to induce canonical NLRP3 inflammasome formation and IL-1β activation (Baker et al., 2015; Ruhl and Broz, 2015).

## HSV1 Manipulation of Pyroptosis Pathways

**FIGURE 3:**
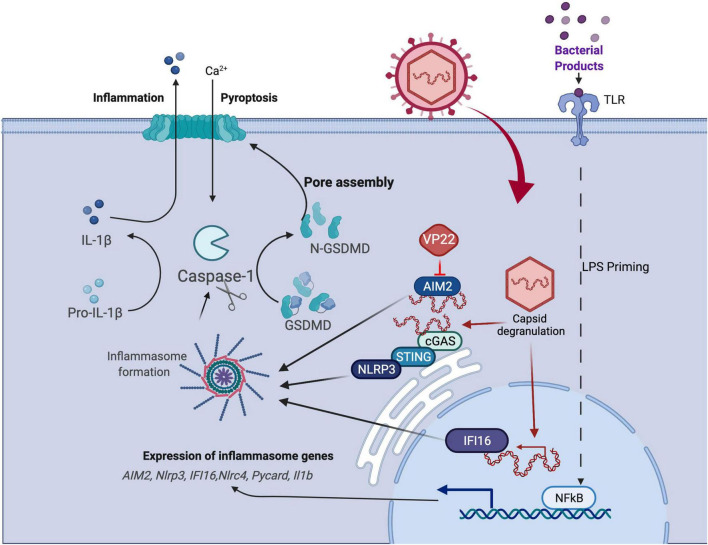
HSV1 manipulation of the pyroptosis pathway. Pyroptosis is defined as GSDM-mediated programmed cell death that is triggered through inflammasome formation and cleavage of pro-caspase 1 to enzymatically active caspase 1 that, in turn, activates GSDM (see text). Studies of virus-mediated pyroptosis rely on *in vitro* models in which cells are primed to upregulate viral sensors and inflammasome components following LPS stimulation followed subsequently by virus infection. HSV1 infection has been shown to manipulate pyroptosis by AIM2 inflammasome blockade *via* virus-encoded VP22, although other suppressors may be involved.

Although HSV1 is a neurotropic virus that predominantly infects epithelial cells and neurons, it exhibits broad cell tropism with variable infectivity rates (Fields et al., 2013). Specifically, this virus can infect macrophages, which are one of the predominant cell types that infiltrate the eye after corneal infection and are crucial to the innate immune response to HSV1 and other viruses (Ghiasi et al., 1995; Horan et al., 2013; Lee and Ghiasi, 2017). During HSV1 infection in macrophages, the cellular ubiquitin proteasome machinery has been reported to degrade the HSV1 nucleocapsid in the cytoplasm, thereby releasing viral DNA into the cytoplasm, which becomes the target for a cytoplasmic DNA sensor (Horan et al., 2013). The AIM2 inflammasome binds specifically to, and is activated by, cytosolic double-stranded DNA. Infection of these cells with MCMV efficiently induced AIM2-dependent inflammasome activation. However, HSV1 infection induces AIM2-independent inflammasome activation either in human monocytic cell line or murine bone marrow derived macrophages (BMDM), indicating HSV1 has evolved a mechanism(s) for evasion of AIM2-dependent inflammasome activation (Rathinam et al., 2010). Kawaguchi group showed that HSV1 tegument protein VP22 inhibits AIM2- dependent inflammasome activation by interacting with AIM2 and prevents its oligomerization (Figure 3). A mutant virus lacking VP22 (HSV1ΔVP22) infection results in diminished viral yields *in vivo*, but HSV1ΔVP22 replication is largely restored in AIM2-deficient mice (Maruzuru et al., 2018). Collectively, their findings reveal a strategy to counteract inflammasome-mediated induction of pro-inflammatory cytokines.

More recently, studies have shown that AIM2 regulates the innate immune sensors pyrin and ZBP1 to drive a form of inflammatory cell death coined PANoptosis (Lee et al., 2021), a PCD phenomenon driven by cytoplasmic multimeric protein complex PANoptosome (Malireddi et al., 2019, 2020; Lee et al., 2021). This form of cell death has been reported to provide host protection during infections with HSV1 (Lee et al., 2021). HSV1 infection of BMDMs triggers a large multi-protein complex containing AIM2, pyrin, ZBP1, ASC, caspase-1, caspase-8, RIPK3, RIPK1 and FADD to drive inflammatory cell death (PANoptosis) (Lee et al., 2021). Interestingly, these studies in BMDM’s suggest that the VP22 which is known to block AIM2 and the downstream death pathways is not sufficient to block cell death in these cells. Further characterization will be required to determine if the inhibition of VP22 has a cell specific effect and if this type of cell death modulates ocular disease. Anyway, PANoptosis does highlight the potential impact of cross-talk between the programmed cell death pathways. Whether this cross-talk between cell death pathways is triggered in human settings still requires further investigation.

Interferon-inducible protein 16 (IFI16), a member of the PYHIN protein, has emerged as a prominent DNA sensor of herpesviruses, capable of recognizing HSV1 DNA in the nuclei of infected cells (Unterholzner et al., 2010; Kerur et al., 2011; Li et al., 2012). Investigations have demonstrated that nuclear IFI16 promotes cytokine expression by signaling to a central cytoplasmic axis in which the endoplasmic reticulum (ER)-resident protein stimulator of interferon genes (STING) engages the serine/threonine TANK-binding kinase 1 (TBK-1). Phosphorylated TBK-1 subsequently phosphorylates the interferon regulatory factor 3 (IRF3), inducing its dimerization and nuclear translocation (Hornung and Latz, 2010). During HSV1, KSHV, and EBV infections, IFI16 was also shown to form supramolecular protein complexes, inflammasomes, which facilitate the maturation of the proinflammatory cytokines, interleukin 1 (IL-1) and IL-18 (Kerur et al., 2011; Ansari et al., 2013; Johnson et al., 2013). Additionally, upon HSV1 infection, IFI16 suppression of viral gene expression was demonstrated to rely in part on facilitating heterochromatinization of the viral genome (Li et al., 2012; Orzalli et al., 2012, 2013).

NLRP3, belongs to the NLR protein family, responds to ATP, bacterial toxins, and microbial components (Mariathasan et al., 2006; Frank and Vince, 2019). NLRP3 inflammasome has also been the most reported in herpes infections, with consequent Caspase-1 and IL-1β activation (Johnson et al., 2013; Wang et al., 2015). A previous report also showed that HSV1 activates NLRP3 inflammasome and therefore plays a protective role against viral immunopathological corneal lesions (Gimenez et al., 2016). Ocular infection of NLRP3-deficient mice led to more-severe and earlier stromal keratitis lesions and had higher angiogenesis scores than did infected wild-type animals, associated with increased early immune response with heightened inflammatory chemokines and cytokines, and elevated recruitment of neutrophils, and increased numbers of CD4+ T cells at later stages of the disease in these Nlrp3^–/–^ animals. This study indicates a regulatory role of NLRP3 in herpetic stromal keratitis pathogenesis. Previous study showed detection of cytosolic DNA by cGAS-STING axis induces a lysosomal cell death (LCD) initiating potassium efflux upstream of NLRP3. A recent report extended the cGAS-STING-LCD-NLRP3 pathway to HSV1 (Wang W. et al., 2020). The study shows STING binds to NLRP3 and promotes the inflammasome activation through two approaches. First, STING recruits NLRP3 and facilitates NLRP3 localization in the endoplasmic reticulum, thereby facilitating the inflammasome formation. Second, STING interacts with NLRP3 and attenuates K48- and K63-linked polyubiquitination of NLRP3, thereby driving inflammasome activation. NLRP3-deficient mice are more susceptible to HSV1 intraperitoneal infection, which elicits weak inflammatory responses, indicating NLRP3 is essential for host defense against HSV1 infection by presumably facilitating IL-1β activation. Whether cGAS-STING axis contributes upstream of NLRP3 during HSV1 ocular infection still requires further exploration.

## Animal Models of HSV1 Corneal Disease

The majority of clinical cases of HSV1 corneal diseases tend to be self-limited without causing permanent vision loss. Upon infection of superficial corneal epithelial cells, virus replication leads to foci of cytopathology that merge to form dendrites that may further merge to form larger geographical ulcers if untreated (Koujah et al., 2019). Of about 20% of these patients, however, the virus will infect the deeper stromal layer of the corneal tissue that may result in the development of HSK. This necrotizing stromal keratitis is an immunopathologic disease caused by an immune response to virus-induced antigens that populate the corneal tissue following virus clearance (Thomas and Rouse, 1997). This immune response is promoted by the formation of new blood vessels (neovascularization) of the normally avascular cornea (Zheng M. et al., 2001; Gimenez et al., 2013). Recurrent episodes of HSK ultimately leads to scarring of the cornea and progressive vision loss over time (Wang L. et al., 2020), a clinical outcome that often leads to corneal transplantation (Garg et al., 2005).

Experimental animal models have provided valuable information on the pathophysiology of HSV1 corneal disease including HSK. Early investigations used an experimental rabbit model of HSK because the rabbit exhibits spontaneous recurrent shedding of virus from the corneal surface following HSV1 infection of the cornea (Webre et al., 2012; Hayashi, 2020) unlike the experimental mouse model of HSK. The relatively large size of the rabbit eye also aided in basic histopathologic studies of primary and recurrent HSV1 disease as well as early pharmaceutical studies focused on the testing of antiviral and anti-inflammatory drugs for the treatment of HSK (Webre et al., 2012; Hayashi, 2020). Of course, a major disadvantage of the experimental rabbit model of HSK has been the lack of immunologic reagents and animals with precise genetic deficiencies to investigate with some precision the immunology and genetics of HSK, respectively.

**FIGURE 4:**
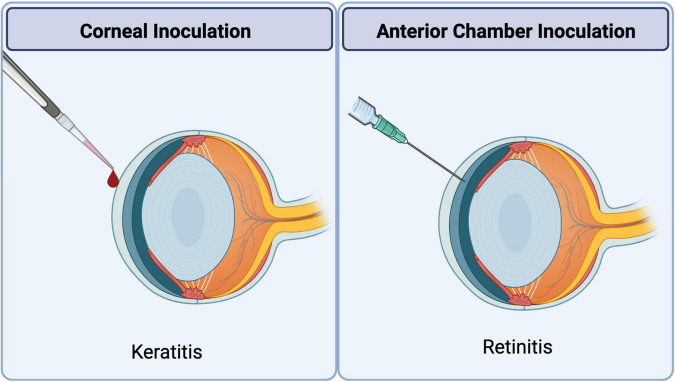
Methods of ocular HSV1 inoculation to induce corneal or retinal disease. Corneal inoculation: Experimental uniocular HSK is induced by topical application of HSV1 onto the corneal surface with or without abrasion depending on the virus strain under investigation. Anterior chamber inoculation: Experimental ARN-like retinal disease is induced in the uninoculated contralateral eye by uniocular injection of HSV1 into the anterior chamber of the ipsilateral eye.

These experimental disadvantages have been overcome through the development of a clinically relevant mouse model of HSV1 corneal disease (Webre et al., 2012). HSK is usually initiated in the mouse eye using most strains of HSV1 through the topical application of the virus onto the corneal surface that has been abraded using a sterile needle (Jiang et al., 2015; Yun et al., 2020; Figure 4). One notable exception is the McKrae strain of HSV1 that does not require corneal abrasion (Matundan et al., 2014) and therefore is often the preferred strain of virus to investigate primary and recurrent HSV1 corneal disease. Characteristic dendritic disease develops within 2 days of inoculation with corneal epithelial cells supporting virus replication. Replicating virus, however, is usually cleared from the cornea within 7 to 8 days after inoculation, although the duration of virus replication varies with dose and strain of virus. It is during this time of virus replication within the corneal tissue that the virus gains access to sensory nerve termini and travels *via* retrograde axonal transport to the neuronal nuclei of the trigeminal ganglion where latency is established. Clearance of infectious virus from the cornea coincides with the cornea regaining clarity, but most previously infected corneas ultimately will develop HSK that is preceded by significant neovascularization accompanied by dramatic lymphocyte infiltration. Experimental HSK in the mouse model of HSV1 corneal disease exhibits an incidence that usually ranges from 60 to 90%. Certain mouse strains, however, are more susceptible to developing experimental HSK (e.g., BALB/c mice) when compared with other mouse strains that show more resistance to HSK development (e.g., C57BL/6 mice). In summary, the experimental mouse model of HSV1 corneal disease progresses from a relatively benign form, with only corneal epithelial involvement, to a severe necrotizing keratitis associated with neovascularization and stromal inflammation by 16 to 20 days after initial virus inoculation.

## Animal Models of HSV1 Retinal Disease

HSV1 can invade the retina of otherwise healthy persons to cause ARN, a severe acute necrotizing retinitis characterized by the sudden onset of vision loss that can lead to permanent blindness (Mueller et al., 2008). Clinical features of ARN include prominent inflammation of the retinal tissue, retinal vasculitis, vitreitis, and retinal detachment. Histopathologic features include full-thickness retinal necrosis with retinal cells exhibiting pathognomonic intranuclear inclusions, massive influx of macrophages, lymphocytes, and polymorphonuclear cells (PMNs), and arteritis (Mueller et al., 2008). A majority of ARN cases have been associated with varicella-zoster virus, another neurotropic alphaherpesvirus (Culbertson, 1996). Nonetheless, HSV1 also has been shown to be a causative virus of this retinal disease in some patients (Matsuo et al., 1986; Lewis et al., 1989; Duker et al., 1990; Ganatra et al., 2000). Both primary and recurrent infection have been identified in ARN cases of HSV1 etiology (Lewis et al., 1989). The precise pathway by which HSV1 gains access to the retina to produce disease remains unclear, but a neural pathway is likely.

The development of animal models that mimic ARN in humans can be traced to 1924 when von Szily (Experimental, 1924) injected HSV into one eye of a rabbit and discovered the development of retinal necrosis within the uninoculated contralateral eye but with relative histopathologic sparing of the retina within the inoculated ipsilateral eye. The von Szily animal model for primary HSV1 retinitis has now been extensively investigated as an animal model for ARN in rabbits and mice for several years. Many laboratories have shown that inoculation of the KOS strain of HSV1 into the anterior chamber of one eye of an immunocompetent BALB/c mouse results in a severe retinal necrosis associated with detection of infectious virus within the uninoculated contralateral eye by 7 to 10 days after infection (Whittum et al., 1984). In sharp contrast, the architecture of the retinal tissue of the inoculated ipsilateral eye remains relatively intact and without detectable infectious virus. Nonetheless, infectious virus can be recovered from brain tissue with peak amounts at 5 to 7 days after infection (Atherton and Streilein, 1987). Importantly, these animals do not display signs or symptoms of clinical encephalitis. Virus tracer studies have shown that virus spreads from the inoculated eye *via* parasympathetic fibers of the suprachiasmatic area of the hypothalamus (Atherton and Streilein, 1987; Margolis et al., 1989; Vann and Atherton, 1991) and ultimate spreads to the contralateral eye from the brain *via* the optic nerve (Vann and Atherton, 1991), findings that have been confirmed using the rabbit model of ARN (Olson et al., 1987). Of clinical significance, magnetic resonance imaging analysis of the brain of one ARN patient with confirmed HSV-1 retinal necrosis also showed abnormalities in the regions of the optic tracts and lateral geniculate ganglia and without development of clinical encephalitis (Vann and Atherton, 1991). Unilateral anterior chamber inoculation of HSV1 strains other than the KOS strain produces bilateral retinitis associated with the development of clinical encephalitis and death in BALB/c mice (Cousins et al., 1989; Figure 4). Subsequent pathogenesis studies have shown that these HSV1 strains enter the brain at least 2 days earlier than the KOS strain, resulting in significant widespread virus replication, and to higher amounts, leading to encephalitis and death at 8 days after intraocular virus infection (Margolis, 1996). It is hypothesized that virus replication at such an earlier time post-infection overwhelms the developing innate immune responses and its effects on limiting virus spread within the brain.

## Role of Cell Death During HSV1 Ocular Infection

Several lines of evidence support a role for apoptosis in limiting HSV replication in the eye, and thereby, protecting it from HSV1 induced pathogenesis. HSV1 infection following anterior chamber inoculation induced apoptosis in the eyes and brain of mice (Qian and Atherton, 2003). HSV1 infection of rabbit corneal epithelial cells induced apoptosis in the underlying keratinocytes (Wilson et al., 1997). Human corneal epithelial cells from patients with ocular HSV1 infection displayed increased apoptosis as well (Miles et al., 2003). Therefore, the results from both animal models and human infections indicate that HSV1 infection leads to apoptosis.

HSV1-infected mice lacking the IFN responsive RNase L gene exhibited more severe HSV1 keratitis and less apoptosis than their wild-type littermates (Zheng X. et al., 2001), suggesting that apoptosis reduces the severity of HSK caused by HSV1 infection. Testing HSK or ARN models directly in either intrinsic apoptosis or extrinsic pathway deficient mice will be more straightforward. Since caspase-8 or FADD-deficient mice is embryonic lethal due to unleashed necroptosis, double knock-out mice including RIPK3 or MLKL knock out will be a valuable tool to investigate the contribution of extrinsic apoptosis in the future.

Since RIPK3 or MLKL deficient mice are viable, such mice would provide insights into the contribution of RIPK3-MLKL mediated necroptosis to HSV1 ocular infection. Our unpublished observations using the HSV1 corneal infection model, higher viral titers were observed in RIPK3 kinase inactive mice, which is lack of RIPK3 kinase mediated necroptosis. This preliminary data suggests RIPK3 kinase-dependent necroptosis restricts viral replication in mice corneal epithelium cells. However, whether this pathway contributes to immune-mediated HSV1 ocular pathogenesis stills need more investigations.

Nlrp3^–/–^ mice was a first *in vivo* model to investigate pyroptosis pathway in the HSK model, in which lesions were largely the consequence of an immunoinflammatory process (Gimenez et al., 2016). Nlrp3^–/–^ mice manifested an early onset, more-severe SK lesions, and angiogenesis when compared with WT animals (Gimenez et al., 2016), suggesting NLRP3 mediated pyroptosis has an immunoregulatory function in HSK pathogenesis.

Another recent study showed corneal infection of mice with the virulent HSV1 strains caused simultaneous expression of the NLRP3, NLRP12, and IFI16 inflammasomes and increased production of the biologically active caspase-1 and pro-inflammatory cytokines IL-1β and IL-18 (Coulon et al., 2019). This intensified inflammatory response was associated with a severe corneal herpetic disease, irrespective of the level of virus replication in the cornea (Coulon et al., 2019). This indicates virulent HSV1 infection leading to pyroptosis, which results in a harmful and overwhelming inflammation that could damage the infected tissue.

## Concluding Remarks

Corneal herpesvirus infection continues to result in blindness. Despite many years of extensive clinical and laboratory investigation, however, basic issues related to the virology, immunology, and pathogenesis of this sight-threatening disease remain unresolved. Among these is a crisp and comprehensive understanding of the basic pathogenic mechanisms and cell death pathways that operate during development of the eye disease caused by HSV1 infection.

After decades of research in host cells especially in human cells, an intricate balance between pro- and anti-cell death signals is established during HSV1 infection. This regulation is no doubt complex, as the number of factors involved in the process continues to grow. Studies imply that there is cell-to-cell variation and even species-specific sensitivity to different cell death pathways. Caspase 8, as the key mediator of necroptosis, also involves in regulation of necroptosis, and pyroptosis (Wilson et al., 1997; Zheng X. et al., 2001; Miles et al., 2003). Conversely, RIPK3 as the key mediator of necroptosis, also contributes to activation of apoptosis (Mandal et al., 2014; Feoktistova and Leverkus, 2015; Coulon et al., 2019). These studies support the cross-talk between cell death pathways during HSV infection. Whether a specific type of cell death pathways is dominant, or a mixture of cell deaths are triggered at the same time during ocular HSV infection is still an open question that requires more investigation.

Rabbit and mouse eye models have been successfully employed to facilitate highly informative studies on ocular HSV1. However, there is limited knowledge of the impact that different cell death pathways have on disease progression. Experiments aimed to directly alter cell death pathways during the development of ocular herpetic disease should clarify this issue.

Trifluridine (TFT) eye drop, acyclovir (ACV) ointment, ganciclovir (GCV) gel, and oral ACV are still the main therapeutic agents for treatment of ocular HSV infection (Roozbahani and Hammersmith, 2018; Valerio and Lin, 2019). Surgical treatment may be necessary to treat complications arising from HSV keratitis (Tuli et al., 2018). The animal models of HSV vaccine are able to reduce HSV keratitis (Dong et al., 2017; Tang et al., 2018; Royer et al., 2019). Other new antivirals are under development. Laboratory discoveries into how HSV interacts with host cell death pathways, and impacts viral production, tissue damage, and host inflammation has deepened our understanding of HSV pathogenesis and opened doors for new therapeutic targets. Overall, the more we understand about the mechanisms of cell death against HSV, the closer we will be to improve therapies against these viruses that cause important burden in humans.

## Author Contributions

HG and RD wrote the manuscript. HK designed the figures and edited the manuscript. EM and RD edited the completed manuscript. All authors contributed to revision and approved the submitted version.

## Conflict of Interest

The authors declare that the research was conducted in the absence of any commercial or financial relationships that could be construed as a potential conflict of interest.

## Publisher’s Note

All claims expressed in this article are solely those of the authors and do not necessarily represent those of their affiliated organizations, or those of the publisher, the editors and the reviewers. Any product that may be evaluated in this article, or claim that may be made by its manufacturer, is not guaranteed or endorsed by the publisher.

## References

[B1] Aglietti R. A. Estevez A. Gupta A. Ramirez M. G. Liu P. S. Kayagaki N. (2016). GsdmD p30 elicited by caspase-11 during pyroptosis forms pores in membranes. *Proc Natl Acad Sci U S A.* 113 7858–7863. 10.1073/pnas.1607769113 27339137 PMC4948338

[B2] Ahmed M. Lock M. Miller C. G. Fraser N. W. (2002). Regions of the herpes simplex virus type 1 latency-associated transcript that protect cells from apoptosis *in vitro* and protect neuronal cells *in vivo*. *J. Virol.* 76 717–729. 10.1128/jvi.76.2.717-729.2002 11752162 PMC136840

[B3] Ansari M. A. Singh V. V. Dutta S. Veettil M. V. Dutta D. Chikoti L. (2013). Constitutive interferon-inducible protein 16-inflammasome activation during Epstein-Barr virus latency I. II, and III in B and epithelial cells. *J. Virol.* 87 8606–8623. 10.1128/JVI.00805-13 23720728 PMC3719826

[B4] Ashkenazi A. Dixit V. M. (1998). Death receptors: signaling and modulation. *Science* 281 1305–1308. 10.1126/science.281.5381.1305 9721089

[B5] Atherton S. S. Streilein J. W. (1987). Two waves of virus following anterior chamber inoculation of HSV-1. *Invest. Ophthalmol. Vis. Sci.* 28 571–579. 3030957

[B6] Aubert M. Blaho J. A. (1999). The herpes simplex virus type 1 regulatory protein ICP27 is required for the prevention of apoptosis in infected human cells. *J. Virol.* 73 2803–2813. 10.1128/JVI.73.4.2803-2813.1999 10074128 PMC104038

[B7] Aubert M. O’Toole J. Blaho J. A. (1999). Induction and prevention of apoptosis in human HEp-2 cells by herpes simplex virus type 1. *J. Virol.* 73 10359–10370. 10.1128/JVI.73.12.10359-10370.1999 10559354 PMC113091

[B8] Baker P. J. Boucher D. Bierschenk D. Tebartz C. Whitney P. G. D’Silva D. B. (2015). NLRP3 inflammasome activation downstream of cytoplasmic LPS recognition by both caspase-4 and caspase-5. *Eur. J. Immunol.* 45 2918–2926. 10.1002/eji.201545655 26173988

[B9] Boucher D. Monteleone M. Coll R. C. Chen K. W. Ross C. M. Teo J. L. (2018). Caspase-1 self-cleavage is an intrinsic mechanism to terminate inflammasome activity. *J. Exp. Med.* 215 827–840. 10.1084/jem.20172222 29432122 PMC5839769

[B10] Bradshaw M. J. Venkatesan A. (2016). Herpes simplex virus-1 encephalitis in adults: pathophysiology, diagnosis, and management. *Neurotherapeutics* 13 493–508. 10.1007/s13311-016-0433-7 27106239 PMC4965403

[B11] Branco F. J. Fraser N. W. (2005). Herpes simplex virus type 1 latency-associated transcript expression protects trigeminal ganglion neurons from apoptosis. *J. Virol.* 79 9019–9025. 10.1128/JVI.79.14.9019-9025.2005 15994795 PMC1168792

[B12] Campbell M. E. Palfreyman J. W. Preston C. M. (1984). Identification of herpes simplex virus DNA sequences which encode a trans-acting polypeptide responsible for stimulation of immediate early transcription. *J. Mol. Biol.* 180 1–19. 10.1016/0022-2836(84)90427-3 6096556

[B13] Carfi A. Willis S. H. Whitbeck J. C. Krummenacher C. Cohen G. H. Eisenberg R. J. (2001). Herpes simplex virus glycoprotein D bound to the human receptor HveA. *Mol. Cell* 8 169–179. 10.1016/s1097-2765(01)00298-2 11511370

[B14] Carpenter D. Hsiang C. Jiang X. Osorio N. BenMohamed L. Jones C. (2015). The herpes simplex virus type 1 (HSV-1) latency-associated transcript (LAT) protects cells against cold-shock-induced apoptosis by maintaining phosphorylation of protein kinase B (AKT). *J. Neurovirol.* 21 568–575. 10.1007/s13365-015-0361-z 26071090 PMC5349860

[B15] Cho Y. S. Challa S. Moquin D. Genga R. Ray T. D. Guildford M. (2009). Phosphorylation-driven assembly of the RIP1-RIP3 complex regulates programmed necrosis and virus-induced inflammation. *Cell* 137 1112–1123. 10.1016/j.cell.2009.05.037 19524513 PMC2727676

[B16] Chuluunbaatar U. Roller R. Feldman M. E. Brown S. Shokat K. M. Mohr I. (2010). Constitutive mTORC1 activation by a herpesvirus Akt surrogate stimulates mRNA translation and viral replication. *Genes Dev.* 24 2627–2639. 10.1101/gad.1978310 21123650 PMC2994037

[B17] Clement C. Tiwari V. Scanlan P. M. Valyi-Nagy T. Yue B. Y. Shukla D. (2006). A novel role for phagocytosis-like uptake in herpes simplex virus entry. *J. Cell Biol.* 174 1009–1021. 10.1083/jcb.200509155 17000878 PMC2064392

[B18] Cookson B. T. Brennan M. A. (2001). Pro-inflammatory programmed cell death. *Trends Microbiol.* 9 113–114. 10.1016/s0966-842x(00)01936-3 11303500

[B19] Coulon P. G. Dhanushkodi N. Prakash S. Srivastava R. Roy S. Alomari N. I. (2019). NLRP3, NLRP12, and IFI16 inflammasomes induction and caspase-1 activation triggered by virulent HSV-1 strains are associated with severe corneal inflammatory herpetic disease. *Front. Immunol.* 10:1631. 10.3389/fimmu.2019.01631 31367214 PMC6644090

[B20] Cousins S. W. Gonzalez A. Atherton S. S. (1989). Herpes simplex retinitis in the mouse. Clinicopathologic correlations. *Invest. Ophthalmol. Vis. Sci.* 30 1485–1494. 2545643

[B21] Culbertson W. W. (1996). “Varicella-zoster virus diseases: posterior segment of the eye,” in *Ocular Infection and Immunity*, eds Pepose J. S. Wilhelmus H. G. Krishna P. V. (St. Louis: Mosby).

[B22] Danial N. N. Korsmeyer S. J. (2004). Cell death: critical control points. *Cell* 116 205–219. 10.1016/s0092-8674(04)00046-7 14744432

[B23] Dillon C. P. Weinlich R. Rodriguez D. A. Cripps J. G. Quarato G. Gurung P. (2014). RIPK1 blocks early postnatal lethality mediated by caspase-8 and RIPK3. *Cell* 157 1189–1202. 10.1016/j.cell.2014.04.018 24813850 PMC4068710

[B24] Ding J. Wang K. Liu W. She Y. Sun Q. Shi J. (2016). Pore-forming activity and structural autoinhibition of the gasdermin family. *Nature* 535 111–116. 10.1038/nature18590 27281216

[B25] Dohner K. Wolfstein A. Prank U. Echeverri C. Dujardin D. Vallee R. (2002). Function of dynein and dynactin in herpes simplex virus capsid transport. *Mol. Biol. Cell* 13 2795–2809. 10.1091/mbc.01-07-0348 12181347 PMC117943

[B26] Dong L. L. Tang R. Zhai Y. J. Malla T. Hu K. (2017). DNA vaccine expressing herpes simplex virus 1 glycoprotein C and D protects mice against herpes simplex keratitis. *Int. J. Ophthalmol.* 10 1633–1639. 10.18240/ijo.2017.11.01 29181304 PMC5686359

[B27] Dufour F. Bertrand L. Pearson A. Grandvaux N. Langelier Y. (2011a). The ribonucleotide reductase R1 subunits of herpes simplex virus 1 and 2 protect cells against poly(I . C)-induced apoptosis. *J. Virol.* 85 8689–8701. 10.1128/JVI.00362-11 21697465 PMC3165841

[B28] Dufour F. Sasseville A. M. Chabaud S. Massie B. Siegel R. M. Langelier Y. (2011b). The ribonucleotide reductase R1 subunits of herpes simplex virus types 1 and 2 protect cells against TNFalpha- and FasL-induced apoptosis by interacting with caspase-8. *Apoptosis* 16 256–271. 10.1007/s10495-010-0560-2 21107701

[B29] Duker J. S. Nielsen J. C. Eagle R. C. Jr. Bosley T. M. Granadier R. Benson W. E. (1990). Rapidly progressive acute retinal necrosis secondary to herpes simplex virus, type 1. *Ophthalmology* 97 1638–1643. 10.1016/s0161-6420(90)32356-4 1965022

[B30] Eaton H. E. Saffran H. A. Wu F. W. Quach K. Smiley J. R. (2014). Herpes simplex virus protein kinases US3 and UL13 modulate VP11/12 phosphorylation, virion packaging, and phosphatidylinositol 3-kinase/Akt signaling activity. *J. Virol.* 88 7379–7388. 10.1128/JVI.00712-14 24741093 PMC4054420

[B31] Experimental A. V. S. (1924). endogenous transmission of infection from bulbus to bulbus. *Klin. Monatsbl. Augenheilkd* 72 593–602.

[B32] Farooq A. V. Valyi-Nagy T. Shukla D. (2010). Mediators and mechanisms of herpes simplex virus entry into ocular cells. *Curr. Eye Res.* 35 445–450. 10.3109/02713681003734841 20465436 PMC2902162

[B33] Feng Y. Daley-Bauer L. P. Roback L. Guo H. Koehler H. S. Potempa M. (2019). Caspase-8 restricts antiviral CD8 T cell hyperaccumulation. *Proc. Natl. Acad. Sci. U.S.A.* 116 15170–15177. 10.1073/pnas.1904319116 31285326 PMC6660791

[B34] Feoktistova M. Leverkus M. (2015). Programmed necrosis and necroptosis signalling. *FEBS J.* 282 19–31. 10.1111/febs.13120 25327580

[B35] Fields B. N. Knipe D. M. Howley P. M. (2013). *Fields Virology*, 6th Edn. Philadelphia, PA: Wolters Kluwer Health/Lippincott Williams & Wilkins.

[B36] Frank D. Vince J. E. (2019). Pyroptosis versus necroptosis: similarities, differences, and crosstalk. *Cell Death Differ.* 26 99–114. 10.1038/s41418-018-0212-6 30341423 PMC6294779

[B37] Galvan V. Roizman B. (1998). Herpes simplex virus 1 induces and blocks apoptosis at multiple steps during infection and protects cells from exogenous inducers in a cell-type-dependent manner. *Proc. Natl. Acad. Sci. U.S.A.* 95 3931–3936. 10.1073/pnas.95.7.3931 9520470 PMC19940

[B38] Ganatra J. B. Chandler D. Santos C. Kuppermann B. Margolis T. P. (2000). Viral causes of the acute retinal necrosis syndrome. *Am. J. Ophthalmol.* 129 166–172. 10.1016/s0002-9394(99)00316-5 10682968

[B39] Garg P. Krishna P. V. Stratis A. K. Gopinathan U. (2005). The value of corneal transplantation in reducing blindness. *Eye (Lond).* 19 1106–1114. 10.1038/sj.eye.6701968 16304591

[B40] Gerster T. Roeder R. G. (1988). A herpesvirus trans-activating protein interacts with transcription factor OTF-1 and other cellular proteins. *Proc. Natl. Acad. Sci. U.S.A.* 85 6347–6351. 10.1073/pnas.85.17.6347 2842768 PMC281967

[B41] Ghiasi H. Wechsler S. L. Kaiwar R. Nesburn A. B. Hofman F. M. (1995). Local expression of tumor necrosis factor alpha and interleukin-2 correlates with protection against corneal scarring after ocular challenge of vaccinated mice with herpes simplex virus type 1. *J. Virol.* 69 334–340. 10.1128/JVI.69.1.334-340.1995 7983727 PMC188580

[B42] Gimenez F. Bhela S. Dogra P. Harvey L. Varanasi S. K. Jaggi U. (2016). The inflammasome NLRP3 plays a protective role against a viral immunopathological lesion. *J. Leukoc Biol.* 99 647–657. 10.1189/jlb.3HI0715-321R 26516184 PMC4831481

[B43] Gimenez F. Suryawanshi A. Rouse B. T. (2013). Pathogenesis of herpes stromal keratitis–a focus on corneal neovascularization. *Prog. Retin. Eye Res.* 33 1–9. 10.1016/j.preteyeres.2012.07.002 22892644 PMC3511644

[B44] Goldstein D. J. Weller S. K. (1988a). Factor(s) present in herpes simplex virus type 1-infected cells can compensate for the loss of the large subunit of the viral ribonucleotide reductase: characterization of an ICP6 deletion mutant. *Virology* 166 41–51. 10.1016/0042-6822(88)90144-4 2842955

[B45] Goldstein D. J. Weller S. K. (1988b). Herpes simplex virus type 1-induced ribonucleotide reductase activity is dispensable for virus growth and DNA synthesis: isolation and characterization of an ICP6 lacZ insertion mutant. *J. Virol.* 62 196–205. 10.1128/JVI.62.1.196-205.1988 2824847 PMC250519

[B46] Green D. R. (1998). Apoptotic pathways: the roads to ruin. *Cell* 94 695–698. 10.1016/s0092-8674(00)81728-6 9753316

[B47] Green D. R. Evan G. I. (2002). A matter of life and death. *Cancer Cell* 1 19–30. 10.1016/s1535-6108(02)00024-7 12086884

[B48] Guo H. Gilley R. P. Fisher A. Lane R. Landsteiner V. J. Ragan K. B. (2018). Species-independent contribution of ZBP1/DAI/DLM-1-triggered necroptosis in host defense against HSV1. *Cell Death Dis.* 9:816. 10.1038/s41419-018-0868-3 30050136 PMC6062522

[B49] Guo H. Omoto S. Harris P. A. Finger J. N. Bertin J. Gough P. J. (2015). Herpes simplex virus suppresses necroptosis in human cells. *Cell Host Microbe* 17 243–251. 10.1016/j.chom.2015.01.003 25674983 PMC4382104

[B50] Hayashi K. (2020). The ultimate terminus of evolutional symbiosis leaves a flaw: blinding herpetic stromal keratitis. *New Front. Ophthalmol.* 6 1–12. 10.1007/978-3-642-35951-4_849-1

[B51] He S. Liang Y. Shao F. Wang X. (2011). Toll-like receptors activate programmed necrosis in macrophages through a receptor-interacting kinase-3-mediated pathway. *Proc. Natl. Acad. Sci. U.S.A.* 108 20054–20059. 10.1073/pnas.1116302108 22123964 PMC3250173

[B52] He S. Wang L. Miao L. Wang T. Du F. Zhao L. (2009). Receptor interacting protein kinase-3 determines cellular necrotic response to TNF-alpha. *Cell* 137 1100–1111. 10.1016/j.cell.2009.05.021 19524512

[B53] Henderson G. Peng W. Jin L. Perng G. C. Nesburn A. B. Wechsler S. L. (2002). Regulation of caspase 8- and caspase 9-induced apoptosis by the herpes simplex virus type 1 latency-associated transcript. *J. Neurovirol.* 8(Suppl. 2) 103–111. 10.1080/13550280290101085 12491160

[B54] Hengartner M. O. (2000). The biochemistry of apoptosis. *Nature* 407 770–776. 10.1038/35037710 11048727

[B55] Honess R. W. Roizman B. (1974). Regulation of herpesvirus macromolecular synthesis. I. Cascade regulation of the synthesis of three groups of viral proteins. *J. Virol.* 14 8–19. 10.1128/JVI.14.1.8-19.1974 4365321 PMC355471

[B56] Horan K. A. Hansen K. Jakobsen M. R. Holm C. K. Soby S. Unterholzner L. (2013). Proteasomal degradation of herpes simplex virus capsids in macrophages releases DNA to the cytosol for recognition by DNA sensors. *J. Immunol.* 190 2311–2319. 10.4049/jimmunol.1202749 23345332 PMC3578088

[B57] Hornung V. Latz E. (2010). Intracellular DNA recognition. *Nat. Rev. Immunol.* 10 123–130. 10.1038/nri2690 20098460

[B58] Huang Z. Wu S. Q. Liang Y. Zhou X. Chen W. Li L. (2015). RIP1/RIP3 binding to HSV-1 ICP6 initiates necroptosis to restrict virus propagation in mice. *Cell Host Microbe* 17 229–242. 10.1016/j.chom.2015.01.002 25674982

[B59] Ibanez F. J. Farias M. A. Gonzalez-Troncoso M. P. Corrales N. Duarte L. F. Retamal-Diaz A. (2018). Experimental dissection of the lytic replication cycles of herpes simplex viruses *in vitro*. *Front. Microbiol.* 9:2406. 10.3389/fmicb.2018.02406 30386309 PMC6198116

[B60] Inman M. Perng G. C. Henderson G. Ghiasi H. Nesburn A. B. Wechsler S. L. (2001). Region of herpes simplex virus type 1 latency-associated transcript sufficient for wild-type spontaneous reactivation promotes cell survival in tissue culture. *J. Virol.* 75 3636–3646. 10.1128/JVI.75.8.3636-3646.2001 11264353 PMC114855

[B61] Jerome K. R. Chen Z. Lang R. Torres M. R. Hofmeister J. Smith S. (2001). HSV and glycoprotein J inhibit caspase activation and apoptosis induced by granzyme B or Fas. *J. Immunol.* 167 3928–3935. 10.4049/jimmunol.167.7.3928 11564811

[B62] Jerome K. R. Fox R. Chen Z. Sears A. E. Lee H. Corey L. (1999). Herpes simplex virus inhibits apoptosis through the action of two genes. Us5 and Us3. *J. Virol.* 73 8950–8957. 10.1128/JVI.73.11.8950-8957.1999 10516000 PMC112926

[B63] Jiang Y. Yin X. Stuart P. M. Leib D. A. (2015). Dendritic cell autophagy contributes to herpes simplex virus-driven stromal keratitis and immunopathology. *mBio* 6:e01426-15. 10.1128/mBio.01426-15 26507231 PMC4626854

[B64] Johnson K. E. Chikoti L. Chandran B. (2013). Herpes simplex virus 1 infection induces activation and subsequent inhibition of the IFI16 and NLRP3 inflammasomes. *J. Virol.* 87 5005–5018. 10.1128/JVI.00082-13 23427152 PMC3624293

[B65] Kaiser W. J. Daley-Bauer L. P. Thapa R. J. Mandal P. Berger S. B. Huang C. (2014). RIP1 suppresses innate immune necrotic as well as apoptotic cell death during mammalian parturition. *Proc. Natl. Acad. Sci. U.S.A.* 111 7753–7758. 10.1073/pnas.1401857111 24821786 PMC4040608

[B66] Kaiser W. J. Upton J. W. Long A. B. Livingston-Rosanoff D. Daley-Bauer L. P. Hakem R. (2011). RIP3 mediates the embryonic lethality of caspase-8-deficient mice. *Nature* 471 368–372. 10.1038/nature09857 21368762 PMC3060292

[B67] Kanukollu V. M. Patel B. C. (2021). *Herpes Simplex Ophthalmicus.* Treasure Island, FL: StatPearls. 32644620

[B68] Kaufmann S. H. Desnoyers S. Ottaviano Y. Davidson N. E. Poirier G. G. (1993). Specific proteolytic cleavage of poly(ADP-ribose) polymerase: an early marker of chemotherapy-induced apoptosis. *Cancer Res.* 53 3976–3985. 8358726

[B69] Kayagaki N. Warming S. Lamkanfi M. Vande Walle L. Louie S. Dong J. (2011). Non-canonical inflammasome activation targets caspase-11. *Nature* 479 117–121. 10.1038/nature10558 22002608

[B70] Kerur N. Veettil M. V. Sharma-Walia N. Bottero V. Sadagopan S. Otageri P. (2011). IFI16 acts as a nuclear pathogen sensor to induce the inflammasome in response to Kaposi Sarcoma-associated herpesvirus infection. *Cell Host Microbe* 9 363–375. 10.1016/j.chom.2011.04.008 21575908 PMC3113467

[B71] Koehler H. Cotsmire S. Langland J. Kibler K. V. Kalman D. Upton J. W. (2017). Inhibition of DAI-dependent necroptosis by the Z-DNA binding domain of the vaccinia virus innate immune evasion protein. E3. *Proc. Natl. Acad. Sci. U.S.A.* 114 11506–11511. 10.1073/pnas.1700999114 29073079 PMC5664489

[B72] Koehler H. Cotsmire S. Zhang T. Balachandran S. Upton J. W. Langland J. (2021). Vaccinia virus E3 prevents sensing of Z-RNA to block ZBP1-dependent necroptosis. *Cell Host Microbe* 29 1266–1276 e5. 10.1016/j.chom.2021.05.009 34192517 PMC9333947

[B73] Koo G. B. Morgan M. J. Lee D. G. Kim W. J. Yoon J. H. Koo J. S. (2015). Methylation-dependent loss of RIP3 expression in cancer represses programmed necrosis in response to chemotherapeutics. *Cell Res.* 25 707–725. 10.1038/cr.2015.56 25952668 PMC4456623

[B74] Koujah L. Suryawanshi R. K. Shukla D. (2019). Pathological processes activated by herpes simplex virus-1 (HSV-1) infection in the cornea. *Cell Mol. Life Sci.* 76 405–419. 10.1007/s00018-018-2938-1 30327839 PMC6349487

[B75] Koyama A. H. Adachi A. (1997). Induction of apoptosis by herpes simplex virus type 1. *J. Gen. Virol.* 78(Pt 11) 2909–2912. 10.1099/0022-1317-78-11-2909 9367378

[B76] Koyama A. H. Miwa Y. (1997). Suppression of apoptotic DNA fragmentation in herpes simplex virus type 1-infected cells. *J. Virol.* 71 2567–2571. 10.1128/JVI.71.3.2567-2571.1997 9032402 PMC191375

[B77] Kristie T. M. Sharp P. A. (1993). Purification of the cellular C1 factor required for the stable recognition of the Oct-1 homeodomain by the herpes simplex virus alpha-trans-induction factor (VP16). *J. Biol. Chem.* 268 6525–6534. 10.1016/s0021-9258(18)53282-8 8454622

[B78] Langelier Y. Bergeron S. Chabaud S. Lippens J. Guilbault C. Sasseville A. M. (2002). The R1 subunit of herpes simplex virus ribonucleotide reductase protects cells against apoptosis at, or upstream of, caspase-8 activation. *J. Gen. Virol.* 83(Pt 11) 2779–2789. 10.1099/0022-1317-83-11-2779 12388814

[B79] Lazebnik Y. A. Kaufmann S. H. Desnoyers S. Poirier G. G. Earnshaw W. C. (1994). Cleavage of poly(ADP-ribose) polymerase by a proteinase with properties like ICE. *Nature* 371 346–347. 10.1038/371346a0 8090205

[B80] Lazebnik Y. A. Takahashi A. Moir R. D. Goldman R. D. Poirier G. G. Kaufmann S. H. (1995). Studies of the lamin proteinase reveal multiple parallel biochemical pathways during apoptotic execution. *Proc. Natl. Acad. Sci. U.S.A.* 92 9042–9046. 10.1073/pnas.92.20.9042 7568069 PMC40920

[B81] Lee D. H. Ghiasi H. (2017). Roles of M1 and M2 macrophages in herpes simplex Virus 1 infectivity. *J. Virol.* 91:e00578-17. 10.1128/JVI.00578-17 28490589 PMC5512262

[B82] Lee S. Karki R. Wang Y. Nguyen L. N. Kalathur R. C. Kanneganti T. D. (2021). AIM2 forms a complex with pyrin and ZBP1 to drive PANoptosis and host defence. *Nature* 597 415–419. 10.1038/s41586-021-03875-8 34471287 PMC8603942

[B83] Lembo D. Brune W. (2009). Tinkering with a viral ribonucleotide reductase. *Trends Biochem. Sci.* 34 25–32. 10.1016/j.tibs.2008.09.008 18990579

[B84] Leopardi R. Roizman B. (1996). The herpes simplex virus major regulatory protein ICP4 blocks apoptosis induced by the virus or by hyperthermia. *Proc. Natl. Acad. Sci. U.S.A.* 93 9583–9587. 10.1073/pnas.93.18.9583 8790373 PMC38471

[B85] Leopardi R. Van Sant C. Roizman B. (1997). The herpes simplex virus 1 protein kinase US3 is required for protection from apoptosis induced by the virus. *Proc. Natl. Acad. Sci. U.S.A.* 94 7891–7896. 10.1073/pnas.94.15.7891 9223283 PMC21525

[B86] Lewis M. L. Culbertson W. W. Post J. D. Miller D. Kokame G. T. Dix R. D. (1989). Herpes simplex virus type 1. A cause of the acute retinal necrosis syndrome. *Ophthalmology* 96 875–878. 10.1016/s0161-6420(89)32823-5 2544841

[B87] Li S. Carpenter D. Hsiang C. Wechsler S. L. Jones C. (2010). Herpes simplex virus type 1 latency-associated transcript inhibits apoptosis and promotes neurite sprouting in neuroblastoma cells following serum starvation by maintaining protein kinase B (AKT) levels. *J. Gen. Virol.* 91(Pt 4) 858–866. 10.1099/vir.0.015719-0 19955563 PMC2888161

[B88] Li T. Diner B. A. Chen J. Cristea I. M. (2012). Acetylation modulates cellular distribution and DNA sensing ability of interferon-inducible protein IFI16. *Proc. Natl. Acad. Sci. U.S.A.* 109 10558–10563. 10.1073/pnas.1203447109 22691496 PMC3387042

[B89] Liesegang T. J. (2001). Herpes simplex virus epidemiology and ocular importance. *Cornea* 20 1–13. 10.1097/00003226-200101000-00001 11188989

[B90] Liu X. Zhang Z. Ruan J. Pan Y. Magupalli V. G. Wu H. (2016). Inflammasome-activated gasdermin D causes pyroptosis by forming membrane pores. *Nature* 535 153–158. 10.1038/nature18629 27383986 PMC5539988

[B91] Liu X. Zou H. Slaughter C. Wang X. (1997). DFF, a heterodimeric protein that functions downstream of caspase-3 to trigger DNA fragmentation during apoptosis. *Cell* 89 175–184. 10.1016/s0092-8674(00)80197-x 9108473

[B92] Malireddi R. K. S. Gurung P. Kesavardhana S. Samir P. Burton A. Mummareddy H. (2020). Innate immune priming in the absence of TAK1 drives RIPK1 kinase activity-independent pyroptosis, apoptosis, necroptosis, and inflammatory disease. *J. Exp. Med.* 217:jem.20191644. 10.1084/jem.20191644 31869420 PMC7062518

[B93] Malireddi R. K. S. Kesavardhana S. Kanneganti T. D. (2019). ZBP1 and TAK1: Master Regulators of NLRP3 Inflammasome/Pyroptosis, Apoptosis, and Necroptosis (PAN-optosis). *Front. Cell Infect. Microbiol.* 9:406. 10.3389/fcimb.2019.00406 31850239 PMC6902032

[B94] Mandal P. Berger S. B. Pillay S. Moriwaki K. Huang C. Guo H. (2014). RIP3 induces apoptosis independent of pronecrotic kinase activity. *Mol Cell* 56 481–495. 10.1016/j.molcel.2014.10.021 25459880 PMC4512186

[B95] Marcocci M. E. Napoletani G. Protto V. Kolesova O. Piacentini R. Li Puma D. D. (2020). Herpes simplex virus-1 in the brain: the dark side of a sneaky infection. *Trends Microbiol.* 28 808–820. 10.1016/j.tim.2020.03.003 32386801

[B96] Margolis T. P. (1996). “Herpes simplex virus diseases: posterior segment of the eye,” in *Ocular Infection and Immunity*, eds Pepose J. S. Wilhelmus H. G. Krishna P. V. (St. Louis: Mosby).

[B97] Margolis T. P. LaVail J. H. Setzer P. Y. Dawson C. R. (1989). Selective spread of herpes simplex virus in the central nervous system after ocular inoculation. *J. Virol.* 63 4756–4761. 10.1128/JVI.63.11.4756-4761.1989 2552151 PMC251112

[B98] Mariathasan S. Weiss D. S. Newton K. McBride J. O’Rourke K. Roose-Girma M. (2006). Cryopyrin activates the inflammasome in response to toxins and ATP. *Nature* 440 228–232. 10.1038/nature04515 16407890

[B99] Maruzuru Y. Ichinohe T. Sato R. Miyake K. Okano T. Suzuki T. (2018). Herpes simplex virus 1 VP22 inhibits AIM2-dependent inflammasome activation to enable efficient viral replication. *Cell Host Microbe* 23 254–265 e7. 10.1016/j.chom.2017.12.014 29447697

[B100] Mashima T. Naito M. Fujita N. Noguchi K. Tsuruo T. (1995). Identification of actin as a substrate of ICE and an ICE-like protease and involvement of an ICE-like protease but not ICE in VP-16-induced U937 apoptosis. *Biochem. Biophys. Res. Commun.* 217 1185–1192. 10.1006/bbrc.1995.2894 8554575

[B101] Matsuo T. Nakayama T. Matsuo N. Koide N. (1986). Immunological studies of uveitis. 1. Immune complex containing herpes virus antigens in four patients with acute retinal necrosis syndrome. *Jpn. J. Ophthalmol.* 30 472–479. 3035258

[B102] Matundan H. Mott K. R. Ghiasi H. (2014). Role of CD8+ T cells and lymphoid dendritic cells in protection from ocular herpes simplex virus 1 challenge in immunized mice. *J. Virol.* 88 8016–8027. 10.1128/JVI.00913-14 24807710 PMC4097799

[B103] Miles D. Athmanathan S. Thakur A. Willcox M. (2003). A novel apoptotic interaction between HSV-1 and human corneal epithelial cells. *Curr. Eye Res.* 26 165–174. 10.1076/ceyr.26.3.165.14899 12815544

[B104] Mocarski E. S. Upton J. W. Kaiser W. J. (2011). Viral infection and the evolution of caspase 8-regulated apoptotic and necrotic death pathways. *Nat. Rev. Immunol.* 12 79–88. 10.1038/nri3131 22193709 PMC4515451

[B105] Mueller N. H. Gilden D. H. Cohrs R. J. Mahalingam R. Nagel M. A. (2008). Varicella zoster virus infection: clinical features, molecular pathogenesis of disease, and latency. *Neurol. Clin.* 26 675–97, viii. 10.1016/j.ncl.2008.03.011 18657721 PMC2754837

[B106] Nagata S. (1997). Apoptosis by death factor. *Cell* 88 355–365. 10.1016/s0092-8674(00)81874-7 9039262

[B107] Newton K. Dugger D. L. Wickliffe K. E. Kapoor N. de Almagro M. C. Vucic D. (2014). Activity of protein kinase RIPK3 determines whether cells die by necroptosis or apoptosis. *Science* 343 1357–1360. 10.1126/science.1249361 24557836

[B108] Oberst A. Dillon C. P. Weinlich R. McCormick L. L. Fitzgerald P. Pop C. (2011). Catalytic activity of the caspase-8-FLIP(L) complex inhibits RIPK3-dependent necrosis. *Nature* 471 363–367. 10.1038/nature09852 21368763 PMC3077893

[B109] Olson R. M. Holland G. N. Goss S. J. Bowers W. D. Meyers-Elliott R. H. (1987). Routes of viral spread in the von Szily model of herpes simplex virus retinopathy. *Curr. Eye Res.* 6 59–62. 10.3109/02713688709020069 3030654

[B110] Orzalli M. H. Conwell S. E. Berrios C. DeCaprio J. A. Knipe D. M. (2013). Nuclear interferon-inducible protein 16 promotes silencing of herpesviral and transfected DNA. *Proc. Natl. Acad. Sci. U.S.A.* 110 E4492–E4501. 10.1073/pnas.1316194110 24198334 PMC3839728

[B111] Orzalli M. H. DeLuca N. A. Knipe D. M. (2012). Nuclear IFI16 induction of IRF-3 signaling during herpesviral infection and degradation of IFI16 by the viral ICP0 protein. *Proc. Natl. Acad. Sci. U.S.A.* 109 E3008–E3017. 10.1073/pnas.1211302109 23027953 PMC3497734

[B112] Perng G. C. Jones C. Ciacci-Zanella J. Stone M. Henderson G. Yukht A. (2000). Virus-induced neuronal apoptosis blocked by the herpes simplex virus latency-associated transcript. *Science* 287 1500–1503. 10.1126/science.287.5457.1500 10688801

[B113] Qian H. Atherton S. (2003). Apoptosis and increased expression of Fas ligand after uniocular anterior chamber (AC) inoculation of HSV-1. *Curr. Eye Res.* 26 195–203. 10.1076/ceyr.26.3.195.14897 12815547

[B114] Rathinam V. A. Jiang Z. Waggoner S. N. Sharma S. Cole L. E. Waggoner L. (2010). The AIM2 inflammasome is essential for host defense against cytosolic bacteria and DNA viruses. *Nat. Immunol.* 11 395–402. 10.1038/ni.1864 20351692 PMC2887480

[B115] Rickard J. A. O’Donnell J. A. Evans J. M. Lalaoui N. Poh A. R. Rogers T. (2014). RIPK1 regulates RIPK3-MLKL-driven systemic inflammation and emergency hematopoiesis. *Cell* 157 1175–1188. 10.1016/j.cell.2014.04.019 24813849

[B116] Rogers C. Fernandes-Alnemri T. Mayes L. Alnemri D. Cingolani G. Alnemri E. S. (2017). Cleavage of DFNA5 by caspase-3 during apoptosis mediates progression to secondary necrotic/pyroptotic cell death. *Nat. Commun.* 8:14128. 10.1038/ncomms14128 28045099 PMC5216131

[B117] Roizman B. Zhou G. (2015). The 3 facets of regulation of herpes simplex virus gene expression: a critical inquiry. *Virology* 47 562–567. 10.1016/j.virol.2015.02.036 25771487 PMC4424108

[B118] Roozbahani M. Hammersmith K. M. (2018). Management of herpes simplex virus epithelial keratitis. *Curr. Opin. Ophthalmol.* 29 360–364. 10.1097/ICU.0000000000000483 29697435

[B119] Royer D. J. Hendrix J. F. Larabee C. M. Reagan A. M. Sjoelund V. H. Robertson D. M. (2019). Vaccine-induced antibodies target sequestered viral antigens to prevent ocular HSV-1 pathogenesis, preserve vision, and preempt productive neuronal infection. *Mucosal. Immunol.* 12 827–839. 10.1038/s41385-019-0131-y 30670763 PMC6462227

[B120] Ruhl S. Broz P. (2015). Caspase-11 activates a canonical NLRP3 inflammasome by promoting K(+) efflux. *Eur. J. Immunol.* 45 2927–2936. 10.1002/eji.201545772 26173909

[B121] Sakamaki K. Inoue T. Asano M. Sudo K. Kazama H. Sakagami J. (2002). *Ex vivo* whole-embryo culture of caspase-8-deficient embryos normalize their aberrant phenotypes in the developing neural tube and heart. *Cell Death Differ.* 9 1196–1206. 10.1038/sj.cdd.4401090 12404118

[B122] Sborgi L. Ruhl S. Mulvihill E. Pipercevic J. Heilig R. Stahlberg H. (2016). GSDMD membrane pore formation constitutes the mechanism of pyroptotic cell death. *EMBO J.* 35 1766–1778. 10.15252/embj.201694696 27418190 PMC5010048

[B123] Shi J. Zhao Y. Wang K. Shi X. Wang Y. Huang H. (2015). Cleavage of GSDMD by inflammatory caspases determines pyroptotic cell death. *Nature* 526 660–665. 10.1038/nature15514 26375003

[B124] Shi J. Zhao Y. Wang Y. Gao W. Ding J. Li P. (2014). Inflammatory caspases are innate immune receptors for intracellular LPS. *Nature* 514 187–192. 10.1038/nature13683 25119034

[B125] Shukla D. Spear P. G. (2001). Herpesviruses and heparan sulfate: an intimate relationship in aid of viral entry. *J. Clin. Invest.* 108 503–510. 10.1172/JCI13799 11518721 PMC209412

[B126] Smith J. S. Robinson N. J. (2002). Age-specific prevalence of infection with herpes simplex virus types 2 and 1: a global review. *J. Infect. Dis.* 186(Suppl. 1) S3–S28. 10.1086/343739 12353183

[B127] Sodeik B. Ebersold M. W. Helenius A. (1997). Microtubule-mediated transport of incoming herpes simplex virus 1 capsids to the nucleus. *J. Cell Biol.* 136 1007–1021. 10.1083/jcb.136.5.1007 9060466 PMC2132479

[B128] Sodroski D. (2021). “Herpes simplex viruses: mechanisms of lytic and latent infection,” in *Fields Virology: DNA Viruses. 2*, ed. Knipe D. M. (Alphen aan den Rijn: Wolters Kluwer, Inc), 235–296.

[B129] Sridharan H. Ragan K. B. Guo H. Gilley R. P. Landsteiner V. J. Kaiser W. J. (2017). Murine cytomegalovirus IE3-dependent transcription is required for DAI/ZBP1-mediated necroptosis. *EMBO Rep.* 18 1429–1441. 10.15252/embr.201743947 28607035 PMC5538628

[B130] Strasser A. O’Connor L. Dixit V. M. (2000). Apoptosis signaling. *Annu. Rev. Biochem.* 69 217–245. 10.1146/annurev.biochem.69.1.217 10966458

[B131] Sun L. Wang H. Wang Z. He S. Chen S. Liao D. (2012). Mixed lineage kinase domain-like protein mediates necrosis signaling downstream of RIP3 kinase. *Cell* 148 213–227. 10.1016/j.cell.2011.11.031 22265413

[B132] Sun L. Wu J. Du F. Chen X. Chen Z. J. (2013). Cyclic GMP-AMP synthase is a cytosolic DNA sensor that activates the type I interferon pathway. *Science* 339 786–791. 10.1126/science.1232458 23258413 PMC3863629

[B133] Sun X. M. MacFarlane M. Zhuang J. Wolf B. B. Green D. R. Cohen G. M. (1999). Distinct caspase cascades are initiated in receptor-mediated and chemical-induced apoptosis. *J. Biol. Chem.* 274 5053–5060. 10.1074/jbc.274.8.5053 9988752

[B134] Takaoka A. Wang Z. Choi M. K. Yanai H. Negishi H. Ban T. (2007). DAI (DLM-1/ZBP1) is a cytosolic DNA sensor and an activator of innate immune response. *Nature* 448 501–505. 10.1038/nature06013 17618271

[B135] Tang R. Zhai Y. Dong L. Malla T. Hu K. (2018). Immunization with dendritic cell-based DNA vaccine pRSC-NLDC145.gD-IL21 protects mice against herpes simplex virus keratitis. *Immunotherapy* 10 189–200. 10.2217/imt-2017-0060 29370719

[B136] Thapa R. J. Ingram J. P. Ragan K. B. Nogusa S. Boyd D. F. Benitez A. A. (2016). DAI Senses Influenza A Virus Genomic RNA and Activates RIPK3-Dependent Cell Death. *Cell Host Microbe* 20 674–681. 10.1016/j.chom.2016.09.014 27746097 PMC5687825

[B137] Thomas J. Rouse B. T. (1997). Immunopathogenesis of herpetic ocular disease. *Immunol. Res.* 16 375–386. 10.1007/BF02786400 9439761

[B138] Tormanen K. Allen S. Mott K. R. Ghiasi H. (2019). The latency-associated transcript inhibits apoptosis *via* downregulation of components of the type i interferon pathway during latent herpes simplex virus 1 ocular infection. *J. Virol.* 93:e00103-19. 10.1128/JVI.00103-19 30814286 PMC6498055

[B139] Tuli S. Gray M. Shah A. (2018). Surgical management of herpetic keratitis. *Curr. Opin. Ophthalmol.* 29 347–354. 10.1097/ICU.0000000000000484 29708929

[B140] Unterholzner L. Keating S. E. Baran M. Horan K. A. Jensen S. B. Sharma S. (2010). IFI16 is an innate immune sensor for intracellular DNA. *Nat. Immunol.* 11 997–1004. 10.1038/ni.1932 20890285 PMC3142795

[B141] Upton J. W. Kaiser W. J. Mocarski E. S. (2010). Virus inhibition of RIP3-dependent necrosis. *Cell Host Microbe* 7 302–313. 10.1016/j.chom.2010.03.006 20413098 PMC4279434

[B142] Upton J. W. Kaiser W. J. Mocarski E. S. (2012). DAI/ZBP1/DLM-1 complexes with RIP3 to mediate virus-induced programmed necrosis that is targeted by murine cytomegalovirus vIRA. *Cell Host Microbe* 11 290–297. 10.1016/j.chom.2012.01.016 22423968 PMC3531981

[B143] Valerio G. S. Lin C. C. (2019). Ocular manifestations of herpes simplex virus. *Curr. Opin. Ophthalmol.* 30 525–531. 10.1097/ICU.0000000000000618 31567695 PMC8900730

[B144] Vann V. R. Atherton S. S. (1991). Neural spread of herpes simplex virus after anterior chamber inoculation. *Invest. Ophthalmol. Vis. Sci.* 32 2462–2472. 1714427

[B145] Wang L. Wang R. Xu C. Zhou H. (2020). Pathogenesis of herpes stromal keratitis: immune inflammatory response mediated by inflammatory regulators. *Front. Immunol.* 11:766. 10.3389/fimmu.2020.00766 32477330 PMC7237736

[B146] Wang S. L. Zhao G. Zhu W. Dong X. M. Liu T. Li Y. Y. (2015). Herpes simplex virus-1 infection or Simian virus 40-mediated immortalization of corneal cells causes permanent translocation of NLRP3 to the nuclei. *Int. J. Ophthalmol.* 8 46–51. 10.3980/j.issn.2222-3959.2015.01.08 25709906 PMC4325240

[B147] Wang W. Hu D. Wu C. Feng Y. Li A. Liu W. (2020). STING promotes NLRP3 localization in ER and facilitates NLRP3 deubiquitination to activate the inflammasome upon HSV-1 infection. *PLoS Pathog.* 16:e1008335. 10.1371/journal.ppat.1008335 32187211 PMC7080238

[B148] Wang X. Li Y. Liu S. Yu X. Li L. Shi C. (2014). Direct activation of RIP3/MLKL-dependent necrosis by herpes simplex virus 1 (HSV-1) protein ICP6 triggers host antiviral defense. *Proc. Natl. Acad. Sci. U.S.A.* 111 15438–15443. 10.1073/pnas.1412767111 25316792 PMC4217423

[B149] Wang Y. Gao W. Shi X. Ding J. Liu W. He H. (2017). Chemotherapy drugs induce pyroptosis through caspase-3 cleavage of a gasdermin. *Nature* 547 99–103. 10.1038/nature22393 28459430

[B150] Webre J. M. Hill J. M. Nolan N. M. Clement C. McFerrin H. E. Bhattacharjee P. S. (2012). Rabbit and mouse models of HSV-1 latency, reactivation, and recurrent eye diseases. *J. Biomed. Biotechnol.* 2012:612316. 10.1155/2012/612316 23091352 PMC3467953

[B151] Whitley R. J. Roizman B. (2001). Herpes simplex virus infections. *Lancet* 357 1513–1518. 10.1016/S0140-6736(00)04638-9 11377626

[B152] Whitley R. Kimberlin D. W. Prober C. G. (2007). “Pathogenesis and disease,” in *Human Herpesviruses: Biology, Therapy, and Immunoprophylaxis*, eds Arvin A. Campadelli-Fiume G. Mocarski E. Moore P. S. Roizman B. Whitley R. (Cambridge: Cambridge University Press). 21348071

[B153] Whittum J. A. McCulley J. P. Niederkorn J. Y. Streilein J. W. (1984). Ocular disease induced in mice by anterior chamber inoculation of herpes simplex virus. *Invest. Ophthalmol. Vis. Sci.* 25 1065–1073. 6469490

[B154] Wilson S. E. Pedroza L. Beuerman R. Hill J. M. (1997). Herpes simplex virus type-1 infection of corneal epithelial cells induces apoptosis of the underlying keratocytes. *Exp. Eye Res.* 64 775–779. 10.1006/exer.1996.0266 9245908

[B155] Yuan J. Kroemer G. (2010). Alternative cell death mechanisms in development and beyond. *Genes Dev.* 24 2592–2602. 10.1101/gad.1984410 21123646 PMC2994033

[B156] Yun H. Yee M. B. Lathrop K. L. Kinchington P. R. Hendricks R. L. St Leger A. J. (2020). Production of the cytokine VEGF-A by CD4(+) T and myeloid cells disrupts the corneal nerve landscape and promotes herpes stromal keratitis. *Immunity* 53 1050–1062 e5. 10.1016/j.immuni.2020.10.013 33207210 PMC7682749

[B157] Zhang D. W. Shao J. Lin J. Zhang N. Lu B. J. Lin S. C. (2009). RIP3, an energy metabolism regulator that switches TNF-induced cell death from apoptosis to necrosis. *Science* 325 332–336. 10.1126/science.1172308 19498109

[B158] Zhang H. Zhou X. McQuade T. Li J. Chan F. K. Zhang J. (2011). Functional complementation between FADD and RIP1 in embryos and lymphocytes. *Nature* 471 373–376. 10.1038/nature09878 21368761 PMC3072026

[B159] Zhang T. Yin C. Boyd D. F. Quarato G. Ingram J. P. Shubina M. (2020). Influenza virus Z-RNAs induce ZBP1-mediated necroptosis. *Cell* 180 1115–1129 e13. 10.1016/j.cell.2020.02.050 32200799 PMC7153753

[B160] Zheng M. Deshpande S. Lee S. Ferrara N. Rouse B. T. (2001). Contribution of vascular endothelial growth factor in the neovascularization process during the pathogenesis of herpetic stromal keratitis. *J. Virol.* 75 9828–9835. 10.1128/JVI.75.20.9828-9835.2001 11559816 PMC114555

[B161] Zheng X. Silverman R. H. Zhou A. Goto T. Kwon B. S. Kaufman H. E. (2001). Increased severity of HSV-1 keratitis and mortality in mice lacking the 2-5A-dependent RNase L gene. *Invest. Ophthalmol. Vis. Sci.* 42 120–126. 11133856

[B162] Zhou G. Galvan V. Campadelli-Fiume G. Roizman B. (2000). Glycoprotein D or J delivered in trans blocks apoptosis in SK-N-SH cells induced by a herpes simplex virus 1 mutant lacking intact genes expressing both glycoproteins. *J. Virol.* 74 11782–11791. 10.1128/jvi.74.24.11782-11791.2000 11090178 PMC112461

[B163] Zychlinsky A. Prevost M. C. Sansonetti P. J. (1992). *Shigella flexneri* induces apoptosis in infected macrophages. *Nature* 358 167–169. 10.1038/358167a0 1614548

